# Establishing a Sustainable Training Program for Laparoscopy in Resource-Limited Settings: Experience in Ghana

**DOI:** 10.5334/aogh.2957

**Published:** 2020-07-30

**Authors:** Mee Joo Kang, Kwabena Breku Apea-Kubi, Kojo Assoku Kwarko Apea-Kubi, Nyabenda-Gomwa Adoula, James Nii Noi Odonkor, Alfred Korbia Ogoe

**Affiliations:** 1 Center for Liver and Pancreatobiliary Cancer, National Cancer Center, Goyang, KR; 2 Department of Surgery, Greater Accra Regional Hospital, Accra, GH; 3 Department of Obstetrics and Gynaecology, Greater Accra Regional Hospital, Accra, GH

## Abstract

**Background:**

Healthcare equipment funded by international partners is often not properly utilized in many developing countries due to low levels of awareness and a lack of expertise. A long-term on-site training program for laparoscopic surgery was established at a regional hospital in Ghana upon request of the Ghana Health Service and local surgeons.

**Objective:**

The authors report the initial 32-month experience of implementing laparoscopic surgery focusing on the trainees’ response, technical independence, and factors associated with the successful implementation of a “new” surgical practice.

**Methods:**

Curricular structure and feedback results of the trainings for doctors and nurses, and characteristics of laparoscopic procedures performed at the Greater Accra Regional Hospital between January 2017 and September 2019 were retrospectively reviewed.

**Findings:**

Comprehensive training including two weeks of simulation workshops followed by animal labs were regularly provided for the doctors. Among the 97 trainees, 27.9% had prior exposure in laparoscopic surgery, 95% were satisfied with the program. Eleven nurses attained professional competency over 15 training sessions where none had prior exposure to laparoscopic surgery. Since the first laparoscopic cholecystectomy in February 2017, 82 laparoscopic procedures were performed. The scope of the surgery was expanded from general surgery (n = 46) to gynecology (n = 33), pediatric surgery (n = 2), and urology (n = 1). The volume of local doctors as primary operators increased from 0% (0/17, February to December 2017) to 41.9% (13/31, January to October 2018) and 79.4% (27/34, November 2018 to September 2019), with 72.5% of the cases being assisted by the expatriate surgeon. There were no open conversions, technical complications, or mortalities. Local doctors independently commenced endoscopic surgical procedures including cystoscopies, hysteroscopies, endoscopic neurosurgeries and arthroscopies.

**Conclusion:**

Sensitization and motivation of the surgical workforce through long-term continuous on-site training resulted in the successful implementation of laparoscopic surgery with a high level of technical independence.

## Introduction

Despite the fact that approximately 25~30% of the global burden of disease is surgical, surgical care in the field of global health is often not prioritized [1]. For decades, the surgical field has been the, “neglected stepchild of global health” [2]. Inequity in the burden of surgical conditions and provision of surgical care in low- and middle-income countries (LMICs) were recently acknowledged [3].

Laparoscopic surgery is now considered a safe, effective, feasible, and cost-effective modality of treatment in resource-limited settings [4]. However, there still is controversy regarding the introduction of laparoscopic surgery in LMICs where open procedures are also considered safe and cost-effective. Moreover, patients do not perceive the advantages of laparoscopic surgery to be as important as in high-income countries [5,6,7]. Nevertheless, Mongolia’s success in its nationwide expansion of laparoscopy revealed its impact in achieving “sustainable progress” for healthcare providers and gaining patients’ trust in healthcare [8]. This implies that the impact of introducing an advanced surgical treatment modality not only improves surgeons’ surgical skills or patients’ direct benefits, but also more importantly, motivates the surgical workforce and consequently strengthens the health system as a whole [3,8].

Healthcare equipment, including equipment for laparoscopic surgery, is heavily funded by international partners in many developing countries. The World Health Organization (WHO) provides guidelines emphasizing on the utilization and maintenance of donated equipment [9]. However, a weak unsustainable funding structure, a low level of awareness, and a hierarchical surgical culture impedes the adoption of “new” technology, while a lack of expertise discourages its use. These are some of the rate-limiting factors to widespread utilization of laparoscopic surgery [10,11].

Consequently, several successful implementation models for laparoscopic surgery are based on the enforcement of infrastructure and long-term education. In Mongolia, a 9-year national training program on laparoscopy increased the performance of laparoscopic cholecystectomy from 2% to 62% [8,12,13]. In Botswana, “contextually appropriate” long-term partnerships with ongoing mentorship was emphasized in their seven-year experience [5].

In Ghana, there have been preliminary reports of laparoscopic cholecystectomy and laparoscopic surgical skills training programs in two teaching hospitals [7,14,15]. However, laparoscopic surgery has not been widely performed in the majority of hospitals in Ghana, although there are a significant number of doctors with prior exposure in laparoscopic surgery, mostly overseas. Moreover, a survey of 1,070 patients revealed that 78% of the patients were willing to pay for laparoscopy, suggesting its socio-economic feasibility in Ghana [16].

Based on the perceived local needs, a multi-year project was launched to strengthen surgical competency and provide quality surgical care by introducing laparoscopic surgery at a regional hospital in Ghana. In this study, the authors report on the initial experience of implementing laparoscopic surgery at the Greater Accra Regional Hospital (GARH). The emphasis is on long term continuous on-site training to achieve improved awareness and technical independence as a basis for a sustainable development.

## Materials and Methods

### Study site

The Greater Accra Regional Hospital is a 420-bed referral government hospital located in the Osu-Klottey sub-Metropolis of the Accra Metropolitan Area in the Greater Accra Region. Among the surgical workforce, there are 17 specialists (six general surgeons, four orthopedic surgeons, three urologists, two neurosurgeons, one plastic surgeon and one pediatric surgeon) and four surgical residents in the Department of Surgery and seven specialists in the Department of Obstetrics & Gynecology. The Department of Surgery performs an average of 1,100 cases a year.

### Project outline

The project, with the objectives of establishing laparoscopic surgery and providing quality surgical care based on continuous on-site training of doctors and nurses at the GARH, was launched in 2016. It was funded by the Korea International Cooperation Agency (KOICA), a government organization under the Ministry of Foreign Affairs of the Republic of Korea, that provides official developmental assistance. In 2016, needs assessment and situation analysis were done through the observation of daily practice and communication with various stakeholders. Essential equipment and instruments were procured and a training curriculum for doctors and nurses was developed based on their main areas of interest, levels of experience, and applicability to daily practice. In 2017, educational workshops for doctors and nurses commenced, and the first laparoscopic cholecystectomy was performed at the GARH in February 2017. Since then, regular educational workshops are presented with the expansion of the scope of laparoscopic surgery across various specialties. The training programs and supervision of laparoscopic surgeries were regularly provided by an expatriate general surgeon specialized in hepato-biliary-pancreatic surgery with 15 years of experience (M.J.K.), stationed in Ghana from March 2016 to February 2020.

### Educational workshops

For the nursing staff, step-by-step workshops including lectures and practical training were provided since January 2017. The workshops covered topics such as the basic principles of minimally invasive surgery, laparoscopy tower, accessories, and hand instruments, with the emphasis on proper handling, cleaning, sterilization, and troubleshooting for long-term maintenance. The training was led by the expatriate surgeon and representatives of medical supply companies (Karl Storz and Olympus) at the beginning, and the nurses who had been trained overseas through the “Doctor LEE Jong-wook Fellowship” later on. After the workshops, continuous on-the-job training was conducted at the operating theater during the real-time procedures. Repetitive refresher courses were provided to address frequent personnel attrition and introduce new procedures led by local surgeons in various specialties. In particular, the workshops before and after the introduction of new procedures were conducted as a team training of doctors and nurses to establish consensus on equipment setting and selection of proper instruments for relevant procedures. There was no formal technical and knowledge-based assessment, but overall performance was subjectively evaluated by the trainer to achieve the final goal of independently assisting in laparoscopic surgery.

In addition, a biomedical engineer and technicians were involved and trained during the installation of the donated equipment to improve the maintenance and repair of the equipment. The representatives and engineers of the medical supply companies led the training, and follow-up technical support was included in the contract. For serious issues requiring overseas inspection, the hospital management was responsible for payment, and a reserve fund was raised by saving a share of patients’ hospital bills.

Comprehensive educational workshops were conducted for the doctors. This included a two-week course of simulation workshops and subsequent animal labs with live pigs (Table 1). At the GARH, a permanent laparoscopic surgical skill simulation laboratory with five box simulators was set up for the workshops and daily independent practice. Each group in the simulation workshop accommodated a maximum of five doctors to ensure immediate and individualized feedback by the facilitator. The training was divided into four sessions of two-hour training to maximize the participants’ concentration and avoid clashes with their working hours. Each session consisted of a 30-minute lecture and 90-minute box simulator training. The training was conducted twice a week to ensure enough time for the trainees to internalize the newly acquired knowledge and skills [17]. A task-based subjective assessment of each simulation training station was done by the facilitator. Although the assessment was not done in a formal and objective format, technical skills in laparoscopic suture with intracorporeal knot tying were evaluated after four sessions of training. The penalty points in Fundamentals of Laparoscopic Surgery (FLS) manual skills guidelines were adapted for evaluation [18]. Considering the high proportion of novices, the coordinated and controlled movement of the instruments to stitch at least three interrupted sutures with appropriate size of bites, closed slit, and secure knots without tearing the skin pad was considered satisfactory, regardless of time and efficiency. The animal lab with one full-day live pig experiment accommodated a maximum of 12 doctors, with three pigs for each group. After the training of each group, the participants anonymously filled in written feedback forms. The doctors’ participation was initially based on invitation or recommendation by the heads of department. Later, most of the participants presented voluntarily. Since 2018, these programs were accredited with Continuing Professional Development (CPD) credits by the Medical and Dental Council of Ghana.

**Table 1:** Curriculum of the training for the doctors.

Category	Items	Contents

**Lectures**	Course introduction	Course overview
	Introduction to laparoscopy	History, indications and contraindications, advantages and limitations, future perspective
	Instruments	The tower, accessories, hand instruments, energy devices
	Port issues	Abdominal access techniques, precautions, review of the guidelines
	Complications	Complications specific to laparoscopic surgery
	Step-by-step operative procedure	Laparoscopic cholecystectomy
		Laparoscopic appendectomy
		Laparoscopic salpingectomy
		Others (according to the specialties of the trainees)
**Simulation**	Peg transfer	Transferring pegs into different posts
	Cutting	Cutting the patterns printed on paper
	Peeling the candies	Peeling off the wrap of the candies
	Intracorporeal suture	Intracorporeal suture on the skin pad
	Knot tying	Intracorporeal knot tying
**Animal lab**	Lecture	Review of the principles
	Pig lab	Laparoscopic cholecystectomy
		Laparoscopic splenectomy
		Laparoscopic oophorectomy
		Laparoscopic uterine horn resection

### Overseas training

Independently from the KOICA project, five doctors (two general surgeons, one orthopedic surgeon, and two gynecologists) and four theater nurses from the GARH were invited to the Republic of Korea for a six-month training program focused on laparoscopic surgery from 2015 to 2019. The training was through the “Doctor LEE Jong-wook Fellowship Program”, organized by the Korea Foundation for International Healthcare (KOFIH), a government organization under the Ministry of Health and Welfare of the Republic of Korea, providing official development assistance.

### Laparoscopic surgery in GARH

Before 2017, laparoscopic surgery was not available at the GARH. Concurrent with ongoing educational workshops, the selection of surgical indications, procedures, and perioperative treatment were discussed routinely on a daily basis at the surgical outpatient clinic and admission ward. Following the first laparoscopic cholecystectomy, opportunities were provided for doctors to acquire surgical skills gradually, starting with holding the telescope to finally becoming a primary operator. Technical support with troubleshooting and peer debriefing were offered in the operating theater to the doctors and nurses regardless of their specialties. “Laparoscopy Bellwether procedures” including laparoscopic cholecystectomy, appendectomy, diagnostic laparoscopy, salpingectomy, oophorectomy, and ovarian cystectomy were the initial targets for laparoscopic surgery, considering the high prevalence of indications as well as the level of expertise of the operators [19]. Locally trained nurses participated in the initial cases, and the overseas trained nurses later interactively implemented their professional competency.

### Data collection and statistical analysis

Feedback forms collected post training and the clinical characteristics of laparoscopic procedures conducted at the GARH between January 2017 and September 2019 were retrospectively reviewed. Demographics of the trainees including age, sex, rank, specialty, institution and prior experience with laparoscopic surgery, and their level of satisfaction with the workshops were analyzed. In the feedback forms, the level of satisfaction was indicated on a five-point Likert scale and those indicating “agree” or “strongly agree” were considered “satisfactory.” The feedback form included open questions asking for the most enjoyable part of the course, major points achieved, and suggestions to improve the quality of the program. Responses to the open questions were analyzed using thematic analysis [20]. The clinical characteristics of laparoscopic procedures including patients’ age, sex, specialties, indications, types of procedure, operation time, complications, post-operative hospital stay, and the number of primary operators were analyzed according to three even-time periods (period one [February–December 2017], period two [January–October 2018], and period three [November 2018–September 2019]). Statistical analyses of the feedback forms and clinical parameters of laparoscopic procedures were performed using Microsoft Excel® 2010 Version 14.0 (Microsoft Corporation, WA, USA). Numerical data that were not normally distributed were reported with median and interquartile ranges (IQR).

This study has been performed in accordance with the Declaration of Helsinki. The Ethics Review Committee of Ghana Health Service advised the authors to get an ethical clearance from the study site, hence this study received institutional permission with exemption of written consent from the Greater Accra Regional Hospital, Accra, Republic of Ghana.

## Results

### Training of the nursing staff for the use and maintenance of the equipment

Theater nurses at the GARH did not have any prior exposure to laparoscopic surgery before 2016. From January 2017 to September 2019, 15 consecutive educational workshops were held. Average of ten nurses participated in each workshop (161 man-days) and 74.5% of them attended the training consecutively. The first five workshops focused on familiarizing theater nurses with the equipment, emphasizing its proper usage, and maintenance. Subsequent training took place repeatedly to maintain awareness in laparoscopic surgery and retain their professional competency with the equipment. By September 2019, 11 theater nurses achieved professional competency by being capable of assisting laparoscopic surgery independently.

### Training of the doctors for minimally invasive surgery

From August 2017 to September 2019, 100 sessions (25 groups) of simulation workshops were performed. Among the trainees, 53.6% (n = 52) were entry-level doctors who were yet to choose their specialties (Table 2). All 78 trainees who attended more than 75% of the course achieved coordinated and controlled movement of the instruments for laparoscopic suturing and knot-tying, regardless of speed or delicacy. Nine of the 20 specialists (45.0%) who participated in the workshops later performed laparoscopic procedures as primary operators.

**Table 2:** Characteristics of the doctors who participated in the training programs.

	Simulation (N = 97)	Animal lab (N = 32)

**Age** (years, median, IQR)	33 (29~38)	35 (32~41)
**Sex** (M:F)	66:31	25:7
**Rank**		
Specialist	20 (20.6%)	12 (37.5%)
Resident	25 (25.8%)	16 (50.0%)
Medical officer	28 (28.9%)	4 (12.5%)
House officer	24 (24.7%)	0
**Specialties**		
General surgery	23 (23.7%)	18 (56.3%)
Gynaecology	17 (17.5%)	12 (37.5%)
Urology	3 (3.1%)	2 (6.2%)
Orthopedic surgery	2 (2.1%)	0
Pre-specialization	52 (53.6%)	0
**Institution**	6 hospitals	6 hospitals
**Prior experience with minimally invasive surgery**		
None	22 (22.7%)	Those who completed the simulation course
Literature	10 (10.3%)	
Basic training including simulation	6 (6.2%)	
Observation of the procedure	23 (23.7%)	
Assisting the procedure	22 (22.7%)	
Primary operator	5 (5.2%)	
Not declared	9 (9.3%)	

**Figure 1:**
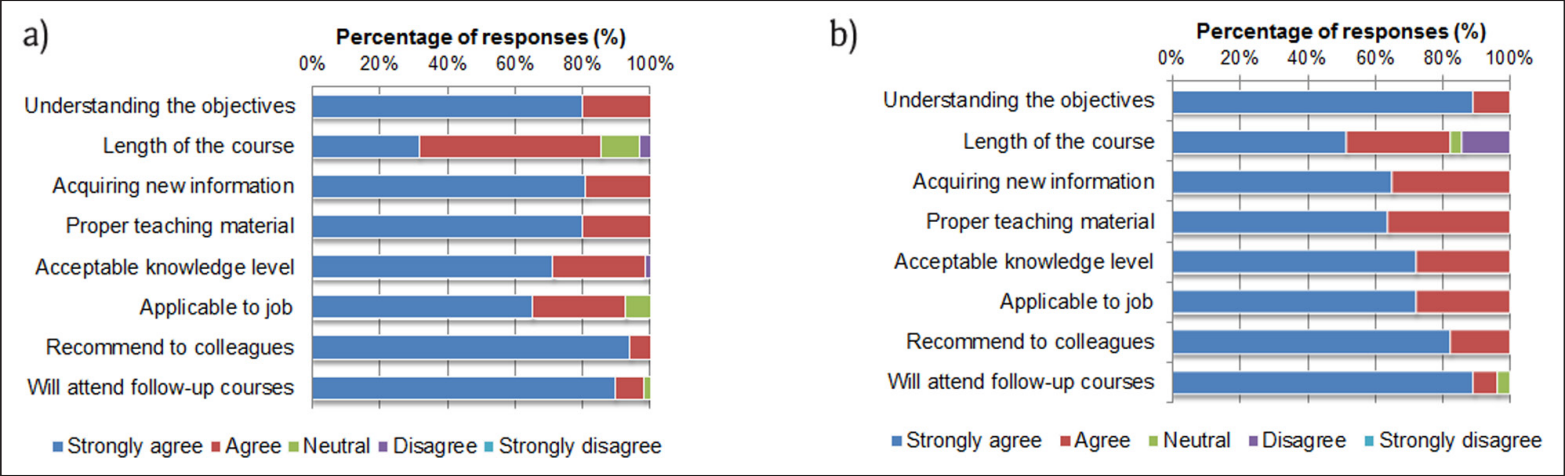
**a)** Overall level of satisfaction in simulation training. **b)** Overall level of satisfaction in animal lab.

After the simulation workshops, 70 trainees (72.2%) filled in the written feedback form. More than 95% of the trainees were satisfied with the contents of the program and lectures. However, the length of the course and applicability to their job showed lower levels of satisfaction (Figure 1a). Feedback results of the lectures and simulation training tasks were satisfactory for more than 95% of the trainees except with regards to the lecture on laparoscopic cholecystectomy (n = 55/61, 90.2%). The most enjoyable part of the course was the hands-on practice (n = 51), structure of the course (n = 15), lecture (n = 12), and interactive teaching method with a small class (n = 11). Major points achieved with the training were knowledge (n = 24), overall understanding and interest (n = 19), and surgical skill (n = 15). The respondents suggested the need for a longer duration of the course with more time to practice (n = 22), an advanced follow-up course with animal or live patient demonstration (n = 10), more video-based teaching material (n = 5), additional subjects specific to their specialties (n = 5), and more diverse simulator training modules (n = 4).

Among those who completed the simulation training, 32 doctors, divided into three groups, participated in the animal laboratory work with live pigs. The response rate for the feedback form was 90.6% (n = 29). More than 95% of the trainees were satisfied with the contents of the course, except the length of the course (82.8%, Figure 1b). Regarding the live pig experiment, the level of satisfaction was relatively lower for gynecologic procedures than for general surgery procedures (laparoscopic oophorectomy 73.3% [11/15], salpingectomy 68.8% [11/16], cholecystectomy 95.2% [20/21], and splenectomy 82.4% [14/17]). The respondents suggested extending the course duration with more time to practice (n = 12) and increasing the number of pigs (n = 2).

### Laparoscopic surgery at GARH

Following the first laparoscopic cholecystectomy at the GARH, 82 laparoscopic procedures were performed between February 2017 to September 2019. The scope expanded from general surgery to other specialties and the proportion of local doctors as primary operators gradually increased (Tables 2 and 4). The expatriate surgeon assisted 72.5% (29/40) of the operations performed by the local doctors as primary operators. Of all the patients, three delayed their discharge because of financial constraints. Two patients had sickle cell disease and one of them was admitted for 15 days for conservative treatment of a post-operative sickle cell crisis. There were no technical complications, open conversions or mortalities.

**Table 3:** Characteristics of the procedures according to time period.

	Period 1 (Feb~Dec 2017)	Period 2 (Jan~Oct 2018)	Period 3 (Nov 2018~Sep 2019)

**Number of operations**	17	31	34
**Age** (years, median, IQR)	40 (31~48)	36 (28~53)	30.5 (27~41)
**Sex** (M:F)	2:17	3:28	4:30
**Specialties**			
General surgery	17 (100%)	18 (58.1%)	11 (32.4%)
Obstetrics & gynecology	0	11 (35.5%)	22 (64.7%)
Pediatric surgery	0	1 (3.2%)	1 (2.9%)
Urology	0	1 (3.2%)	0
**Primary operator**			
Local	0	13 (41.9%)	27 (79.4%)
Expatriate	17 (100%)	18 (58.1%)	7 (20.6%)
**Operation time** (minutes, median, IQR)	45 (30~60)	60 (45~120)	60 (40~150)
**Open conversion**	0	0	0
**Complications**	1 (5.9%)^a^	0	1 (2.9%)^b^
**Postoperative hospital stay** (days, median, IQR)	1 (1~3)	1 (1~2)	1 (1~2)
>5 days	2 (11.8%)	0	2 (5.9%)

^a^ Paralytic ileus; ^b^ Sickle cell crisis.

**Table 4:** Indications and laparoscopic procedures according to time period.

		Total (n = 82)	Period 1 (Feb~Dec 2017)	Period 2 (Jan~Oct 2018)	Period 3 (Nov 2018~Sep 2019)

**Indications**					
General surgery	Gallbladder stone related disease	37	15 (88.2%)	12 (66.7%)	10 (90.9%)
	Appendicitis	6	2 (11.8%)	3 (16.7%)	1 (9.1%)
	Appendicitis with gallbladder stone	3	0	3 (16.7%)	0
	Subtotal	46	17	18	11
Obstetrics & gynecology	Infertility	10	0	3 (27.3%)	7 (31.8%)
	Ovarian cyst	9	0	4 (36.4%)	5 (22.7%)
	Ectopic pregnancy	5	0	2 (18.2%)	3 (13.6%)
	Uterine myoma	4	0	0	4 (18.2%)
	Chronic pelvic pain	2	0	0	2 (9.1%)
	Endometriosis	2	0	2 (18.2%)	0
	Tubo-ovarian abscess	1	0	0	1 (4.5%)
	Subtotal	33	0	11	22
Pediatric surgery	Undescended testis	2	0	1 (100%)	1 (100%)
Urology	Renal cyst	1	0	1 (100%)	0
**Procedures**					
General surgery	Laparoscopic cholecystectomy	37	15 (88.2%)	12 (66.7%)	10 (90.9%)
	Laparoscopic appendectomy	6	2 (11.8%)	3 (16.7%)	1 (9.1%)
	Laparoscopic appendectomy with cholecystectomy	3	0	3 (16.7%)	0
	Subtotal	46	17	18	11
Obstetrics & gynecology	Diagnostic laparoscopy	12	0	5 (45.5%)	7 (31.8%)
	Laparoscopic salpingectomy/salpingo-oophorectomy/oophorectomy	7	0	2 (18.2%)	5 (22.7%)
	Laparoscopic cornual wedge resection	1	0	1 (9.1%)	0
	Laparoscopic ovarian cystectomy	7	0	3 (27.3%)	4 (18.2%)
	Laparoscopic myomectomy	3	0	0	3 (13.6%)
	Laparoscopy-assisted vaginal hysterectomy	2	0	0	2 (9.1%)
	Total laparoscopic hysterectomy with bilateral salpingo-oophorectomy	1	0	0	1 (4.5%)
	Subtotal	33	0	11	22
Pediatric surgery	Laparoscopic exploration of undescended testis	2	0	1 (100%)	1 (100%)
Urology	Laparoscopic kidney cyst marsupialization	1	0	1 (100%)	0

Between 2018 and 2019, 75.9% (22/29) of all cholecystectomies, 1.7% (7/418) of all appendectomies, 14.0% (7/50) of all ovarian cystectomies, and 1.6% (5/314) of surgeries for ectopic pregnancy at the GARH were performed laparoscopically.

### Other endoscopic surgical procedures at GARH

Local doctors who were trained for other endoscopic surgical procedures initiated their practice utilizing the laparoscopy tower and experienced theater nurses. The neurosurgeon first performed endoscopic third ventriculostomy (n = 3) in 2017. Since 2018, the gynecologists performed diagnostic hysteroscopy (n = 5), hysteroscopic myomectomy (n = 2), and polypectomy (n = 1). The urologists also introduced diagnostic (n = 70) and therapeutic cystoscopic procedures (n = 79, including transurethral resection of the prostate or bladder, transurethral vaporization of the prostate, double-J stent insertion, etc.) in the same year. Later, the orthopedic surgeon introduced diagnostic arthroscopy (n = 2) in 2019. Besides intermittent technical assistance for the equipment from the expatriate surgeon, all of the procedures were performed by local surgeons.

## Discussion

In this project, long-term continuous on-site training of the relevant stakeholders was the core strategy to implement laparoscopic surgery at a regional hospital in Ghana. Over time, those who had prior experience but were not routinely performing laparoscopic surgery and those without prior experience, were both able to perform laparoscopic surgery with minimum risks. A steep increase in primary operators among local doctors reflected the progression of technical independence, which is essential for long-term sustainability but difficult to attain over a short time period [21]. Furthermore, endoscopic surgical procedures in neurosurgery, gynecology, urology, and orthopedic surgery were phased in by local doctors, supported by the trained nurses and functional equipment.

There were several factors contributing towards the successful training. First, inclusive training for the doctors, nurses, biomedical engineers, and private sector medical supply companies increased awareness, motivation, and understanding in each other’s role in the field of laparoscopic surgery [4,8,19]. Importantly, training for senior doctors and nurses had a favorable influence on the hierarchical culture, and the former supported their junior colleagues to participate actively in the subsequent workshops [17,19]. Second, clinical collaboration with technical support at the operating theater and in daily clinical practice encouraged doctors to take up the challenge with a minimal risk of technical complications or open conversions [22]. In particular, practical training adjusted to the level of the participants’ experience and clinical applicability was highly prioritized in the locally developed curriculum for the doctors and nurses [5]. Additionally, the small class size of each training promoted individualized, interactive teaching with timely feedback and demonstration to maximize acquisition of skills and knowledge [17]. Third, the doctors were strongly motivated, which is crucial for successful skills training [17,23]. Most of the doctors enrolled in the program voluntarily with remarkable internal motivation, and 53.6% of the participants were entry-level doctors who were yet to determine their specialties. Additionally, the participants highly appreciated several external motivating factors, such as the use of a box simulator or opportunities for participating in an advanced follow-up course with a live pig experiment. Fourth, there was an on-site dedicated surgeon managing the program with a properly equipped simulation laboratory throughout the period [17]. The permanent simulation laboratory was also located next to the operating theater to increase accessibility, and the dedicated faculty ensured efficient utilization of space and equipment. Consumables for the training were designed with inexpensive material, such as papers with printed patterns, candies, or silk suture to minimize financial restraints. Finally, there was the synergistic effect of the supporting foreign governmental organizations in terms of donating equipment, continuous on-site training, and overseas invitational training. Unlike instances where limited resources impede maintenance and the development of the skills acquired abroad, the doctors and nurses who completed the overseas training were able to apply their knowledge and skills in their daily practice at the GARH. Moreover, they played a key role in facilitating the training workshops or initiating “new” procedures after their overseas training to motivate and enroot laparoscopic surgery at the institution.

However, there were several practical barriers to implementing laparoscopic surgery. First, during the transition of the funding source from the foreign donor to the local organization, instability of financial resources and limited autonomy in centralized financial management of the government hospital endangered the continuity of the clinical practice [19]. In fact, laparoscopic surgery was a reliable resource of extra revenue to the hospital because initial investment costs were minimized through the use of donated equipment. Moreover, the hospital earned immediate income compared to delayed reimbursement for procedures that are covered by national health insurance. Nevertheless, it was difficult to create a virtuous cycle such as by the offering of monetary incentives to healthcare workers or subsidies to patients, or expeditious procurement of the consumables. Enhanced awareness and understanding in laparoscopic surgery by the administration members would be crucial in facilitating the administrative process [3]. Second, a shortage of human resources and a high attrition rate of nursing staff not only hindered maintenance and the development of their knowledge and skills, but also increased the workload of the skilled nurses, which resulted in a vicious cycle of higher rates of attrition. Although education and training opportunities have motivating effects, monetary and non-monetary incentives including career development or recognition by the management team should be introduced to retain skilled personnel in the long term as well as to encourage active participation in various clinical settings [24]. Third, there was a limited number of cases available for laparoscopic surgery, making it difficult to get over the learning curve in a short period. Although appendicitis or ectopic pregnancy were good indications for the necessity of laparoscopic surgery, less than 2% of them were treated laparoscopically because of a shortage of personnel and a lack of cooperation among the surgical workforce for emergency operations. Most of the non-physician anesthetic staff were not actively involved in the stakeholder training because physician anesthesiologists were supposed to participate in laparoscopic procedures. This later turned out to be a major setback. Therefore, participation of a comprehensive group of stakeholders should be ensured throughout the course [19]. However, the aforementioned “missed” indications for laparoscopic surgery imply potential room for scaling up in the future. Fourth, there was a significant economic barrier to the patients’ choice [6,7]. Currently, laparoscopic surgery is not covered by national health insurance in Ghana. Although only the minimum cost of additional consumables was added to the tariff of open procedure at the GARH, a significant proportion of patients considered the advantages of laparoscopic surgery, such as less scarring, rapid recovery, and reduced hospital stay less important than the cost difference. Finally, although the doctors showed remarkable motivation individually, they were often frustrated by the aforementioned systemic obstacles. Therefore, apart from strengthening the managerial aspect and entire surgical workforce, building a local collaborative group in laparoscopic surgery to encourage each other and making opportunities to update their knowledge and skills is necessary.

In sub-Saharan Africa, various training programs for basic laparoscopic surgical skill have been implemented in partnership with high-income countries. In this context, short-term training was considered insufficient to acquire technical skills in a three-day FLS course in Botswana [25]. On the other hand, minimal retention of laparoscopic surgical skills at a one-year follow-up after training has been observed in Kenya due to limited opportunities for practical application [26]. However, the case volume doubled after the repetition of a two-day training program in combination with live surgery for surgical residents in Ethiopia, where the faculty surgeons had already been performing laparoscopies [27]. Therefore, both the training period or methods and the accumulated experience in daily practice are essential in training programs for laparoscopy. In this study, the training was not a one-off exercise but continued on-site and was combined with simulation training, animal experiments, and on-the-job training at the operating theater. The feedback results revealed that even after both simulation and animal laboratory work, the trainees were not confident with their newly acquired surgical skills. It is obvious that they need exposure and involvement in more cases at the operating theater to gain practical experience in both surgical skills and situation handling in a variety of clinical scenarios.

This study has several limitations. First, the training was biased to general surgery, having only one dedicated general surgeon for the entire program. As evident from the feedback, additional topics specific to the participants’ specialties should be provided and more experts from various specialties should be involved in the program. The trainees also requested for longer duration of simulation training with more frequent animal experiment. Although the simulation laboratory was open for independent practice, most of the participants found it difficult to visit the place in their spare time. Therefore, incorporation of the program into their formal training curriculum with dedicated time for skills training would be required to ensure sustainability [4,10,17]. In addition, there was no formal technical and knowledge-based assessment apart from task-based subjective assessment. Moreover, the achievement in the cognitive and manual skills of the doctors could not be accredited by professional or academic societies, as Ghana has no system for laparoscopic or endoscopic skill qualification. A qualification system needs to be set up to encourage doctors to achieve and maintain their knowledge and skills. Second, the scale of the study period and population were too small to evaluate the impact of laparoscopic surgery on the health system as in Mongolia [13]. Follow-up studies are needed to investigate long-term sustainability of the training program and its spill-over effect. Third, the cost-effectiveness of laparoscopic surgery by utilizing donated equipment could not be evaluated. As shown in a cost-effectiveness analysis of laparoscopic versus open cholecystectomy at Rwanda Military Hospital, laparoscopic surgery may not be cost-effective at commonly accepted willingness-to-pay thresholds with low case volume in LMICs, but laparoscopic surgery can be cost-effective “in settings where machinery has already been donated” [28].

In conclusion, long-term continuous on-site training with clinical collaboration was effective in implementing laparoscopic surgery, through the sensitization and motivation of the surgical workforce. Donated equipment was utilized through the synergistic effects of the supporting foreign governmental organizations and local authorities. Although there were limitations in terms of case volume, high-level technical independence and the active participation of entry-level doctors would increase sustainability of laparoscopic surgery. Long-term structured training and maintenance of motivated surgical workforce should be considered as core strategies to implement new surgical practice.
